# Supplementary material for: Impact of Sn-doping on the optoelectronic properties of zinc oxide crystal: DFT approach
Source: PLoS One. 2024 Jan 2;19(1):e0296084. doi: 10.1371/journal.pone.0296084 (PMC10760716; doi:10.1371/journal.pone.0296084)
Supplement: Attachment — Submitted filename: report .docx [file pone.0296084.s002.docx]

Comments

**Dear Editor of Journal PLOS ONE.**

This manuscript reported the Impact of Sn-Doping on the Optoelectronic Properties of Zinc

Oxide Crystal: DFT Approach. However, the manuscript is well written and the results are not explained properly. Hence, the manuscript needs more modifications in the present state. Therefore, a major revision should be made before its publication.

Other comments:

-The Abstract is poor language so authors must rewrite.

-Replace First to the first.

-Delete Basically,

-Put and before absorption coefficient;

-detailed out in this study. Delete out

-doping in ZnO play replace to : doping in ZnO plays

-Authors should include their finding in the abstract

Kindly, support the application of ZnO-based in scientific and industrial fields. Refe..., *J. Environ. Manage.* **270**, 110816 (2020); *ECS J. Solid State Sci. Technol.* **10**, 063007 (2021),; *Int. J. Environ. Sci. Technol.* **17**, 4481–4494 (2020); <https://doi.org/10.1007/s12633-022-01886-2>

<https://doi.org/10.1149/2162-8777/ac07f9>

<https://doi.org/10.1007/s12633-021-01618-y>

<https://doi.org/10.1007/s10854-022-08158-0>

10.21608/EJCHEM.2021.87118.4214

<https://doi.org/10.1088/1402-4896/ac119e>

<https://doi.org/10.1007/s10854-021-07182-w>

- Authors must give reasons for their finding (The undoped ZnO show the least refractive index, whereas as the Sn (3 atom) doped ZnO crystal shows the highest refractive index)?

- Check the English grammar of the text.

Regards
