# Supplementary material for: Impact of Sn-doping on the optoelectronic properties of zinc oxide crystal: DFT approach
Source: PLoS One. 2024 Jan 2;19(1):e0296084. doi: 10.1371/journal.pone.0296084 (PMC10760716; doi:10.1371/journal.pone.0296084)
Supplement: Attachment — Submitted filename: Response_to_reviewers_plos_one.docx [file pone.0296084.s003.docx]

**Original Manuscript ID:** PONE-D-23-35532

**Original Article Title: “Impact of Sn-Doping on the Optoelectronic Properties of Zinc Oxide Crystal: DFT Approach”**

**To**

The Editor

PLOS ONE

**Re:** Response to reviewers

Dear Editor,

Thank you for allowing a resubmission of our manuscript, with an opportunity to address the reviewers’ comments.

We are uploading (a) our point-by-point response to the reviewer comments (response to reviewers), (b) an updated revised manuscript with yellow highlighting indicating changes, and (c) a clean updated manuscript without highlights (*Main Manuscript).*

Kindly accept our revised manuscript for further processing.

Best regards,

**Manoj Kumar** et al.

**Reviewer#1, Comment # 1:** **The title is appropriate for the content, the abstract is not convenient and should be modified to focus on the obtained results. - It's better to change the title with remove the word: Basically, out.**

**Author response:** We would like to thank the learned reviewer for his valuable comments which helped to improve the manuscript.

**Author action:** We updated the grammatical errors and typo mistake in several places.

**Reviewer#1, Comment # 2:** **Rewrite the abstract: indicate the most interesting results, avoid repetitions and check the English grammar.**

**Author response:** We would like to thank the learned reviewer for his valuable comments which helped to improve the manuscript.

**Author action:** We updated the grammatical errors and typo mistake in several places and abstract has been re-written. We have incorporated the response at page 1.

**Reviewer#1, Comment # 3:** **The novelty of the work needs to be declared in the introduction part**

**Author response:** As per the suggestion of reviewer, authors have also included structural analysis as shown in Fig 4 in the revised manuscript along with optical investigation to enhance the novelty of this work. However, the effect of tin (Sn) doping of ZnO crystal on its optical and structural characteristics have not been much explored to the author’s best of knowledge. Thus, the Sn-doped ZnO crystal's potential in optoelectronic applications has been assessed by demonstrating and explaining how to tune its optical and structural properties.

**Author action:** We have incorporated the response at page 9.

**Reviewer#2, Concern # 4:** **The whole manuscript should be revised for grammatical and writing errors.**

**Author response:** We would like to thank the learned reviewer for his valuable comments which helped to improve the manuscript.

**Author action:** We updated the grammatical errors and typo mistake in several places.

**Reviewer#1, Comment # 5** :**The introduction and results are somewhat interesting but needs to be specific and focused on the finding and work novelty.**

**Author response:** As per the suggestion of reviewer, authors have highlighted the novelty of work in introduction section.

**Author action:** We have incorporated the response at page 2.

**Reviewer#1, Comment #6:** **Authors must compare their results with the previously reported.**

**Author response:** As per the suggestion of reviewer, authors have compared the obtained results with previously reported.

**Author action:** We have incorporated the response at page 10.

**Reviewer#2, Comment # 1: The Abstract is poor language so authors must rewrite.**

**Author response:** We would like to thank the learned reviewer for his valuable comments which helped to improve the manuscript.

**Author action:** We updated the grammatical errors and typo mistake in several places and abstract has been re-written. We have incorporated the response at page 1.

**Reviewer#2, Comment # 2:**  **Replace First to the first.**

**Author response:** We have addressed this comment by the learned reviewer by removing unnecessary words and made more concise.

**Author action:** We have incorporated the response at page 1.

**Reviewer#2, Comment # 3:** **Delete Basically.**

**Author response:** We have addressed this comment by the learned reviewer by removing unnecessary words and made more concise.

**Reviewer#2, Comment # 4:** **Put and before absorption coefficient.**

**Author response:** We have addressed this comment by the learned reviewer by incorporating the some more discussion in conclusion section.

**Author action:** We updated the manuscript and highlighted in page no. 20.

**Reviewer#2, Comment # 5:**  **detailed out in this study. Delete out**

**Author response:** We have addressed this comment by the learned reviewer by removing unnecessary words and made more concise.

**Reviewer#2, Comment #6:**  **doping in ZnO play replace to : doping in ZnO plays**

**Author response:** We have addressed this comment by the learned reviewer by removing unnecessary words and made more concise.

**Reviewer#2, Comment #7:**  **Authors should include their finding in the abstract**

**Author response:** We would like to thank the learned reviewer for his valuable comments which helped to improve the manuscript.

**Author action:** We updated the grammatical errors and typo mistake in several places and abstract has been re-written. We have incorporated the response at page 1.

**Reviewer#2, Comment # 8:**  **Kindly, support the application of ZnO-based in scientific and industrial fields. Refe..., J. Environ. Manage. 270, 110816 (2020); ECS J. Solid State Sci. Technol. 10, 063007 (2021),; Int. J. Environ. Sci. Technol. 17, 4481–4494 (2020); https://doi.org/10.1007/s12633-022-01886-2**

**https://doi.org/10.1149/2162-8777/ac07f9**

**https://doi.org/10.1007/s12633-021-01618-y**

**https://doi.org/10.1007/s10854-022-08158-0**

**10.21608/EJCHEM.2021.87118.4214**

**https://doi.org/10.1088/1402-4896/ac119e**

**https://doi.org/10.1007/s10854-021-07182-w**

**Author response:** As per the suggestion of the learned reviewer, aforementioned references have been included in the revised manuscript

**Author action:** We updated the manuscript and highlighted in reference section at page 14.

**Reviewer#2, Comment # 9:**  **Authors must give reasons for their finding (The undoped ZnO show the least refractive index, whereas as the Sn (3 atom) doped ZnO crystal shows the highest refractive index)?**

**Author response:** As per the suggestion of the learned reviewer, the reasons for increase in refractive with Sn-doping has been discussed and now included in the revised manuscript.

**Author action:** We updated the suggestion of the learned reviewer at page 7.

**Reviewer#2, Comment #10:**  **- Check the English grammar of the text**

**Author response: Author response:** We have addressed this comment by removing unnecessary words, made more concise and also checked the enitre manuscript thoroughly for any grammatical mistakes.
